# DNA methylome profiling of blood to identify individuals in a pair of monozygotic twins

**DOI:** 10.1007/s13258-023-01396-4

**Published:** 2023-05-17

**Authors:** Jae-Yoon Kim, Hwan Young Lee, So-Yeon Lee, Seon-Young Kim, Jong-Lyul Park, Soong Deok Lee

**Affiliations:** 1 Personalized Genomic Medicine Research Center, Korea Research Institute of Bioscience & Biotechnology, Daejeon, 34141 Korea; 2 Department of Forensic Medicine, Seoul National University College of Medicine, Seoul, 03080 Korea; 3 Institute of Forensic and Anthropological Science, Seoul National University College of Medicine, Seoul, 03080 Korea; 4 Graduate School of New Drug Discovery and Development, Chungnam National University, Daejeon, 34134 Korea; 5 Aging Convergence Research Center, Korea Research Institute of Bioscience & Biotechnology, Daejeon, 34141 Korea

**Keywords:** DNA methylation, Identical twin, Forensic, EPIC BeadChip, Biomarker

## Abstract

**Background:**

Short tandem repeat (STR) markers cannot be used to distinguish between genetically identical monozygotic (MZ) twins, causing problems in a case with an MZ twin as a suspect. Many studies have shown that in older MZ twins, there are significant differences in overall content and genomic distribution of methylation.

**Objective:**

In this study, we analyzed the DNA methylome profile of blood to identify recurrent differentially methylated CpG sites (DMCs) to discriminate between MZ twins.

**Methods:**

Blood samples were collected from 47 paired MZ twins. We performed the DNA methylation profiling using the HumanMethylation EPIC BeadChip platform and identified recurrent DMCs between MZ twins. Then, Kyoto Encyclopedia of Genes and Genomes (KEGG), Gene Ontology (GO), and motif enrichment analyses were performed to reveal the biological functions of recurrent DMCs. We collected DNA methylome data from the Gene Expression Omnibus (GEO) public database to verify the recurrent DMCs between MZ twins.

**Results:**

We identified recurrent DMCs between MZ twin samples and observed that they were enriched in immune-related genes. In addition, we verified our DMCs in a public dataset.

**Conclusion:**

Our results suggest that the methylation level at recurrent DMCs between MZ twins may serve as a valuable biomarker for identification of individuals in a pair of MZ twins.

## Introduction

Identifying individuals from a pair of monozygotic (MZ) twins is important in the forensic sciences, particularly for cases concerning an MZ twin as a suspect or alleged parent. Unfortunately, short tandem repeat (STR) markers cannot distinguish MZ twins because they share an identical genomic DNA sequence. Although several studies have reported that MZ twins have a very small degree of genetic differences, such as single-nucleotide polymorphisms (SNPs) (Weber-Lehmann et al. 2014) or copy number variations (CNVs) (Abdellaoui et al. 2015; McRae et al. 2015), these sequence differences are sparse.

DNA methylation, which occurs at the 5’-position of cytosine in CpG dinucleotides, is involved in regulating gene expression (Moore et al. 2013). As individual DNA methylation levels are uniquely changed by several factors such as environment or disease (Fraga et al. 2005; Planterose Jimenez et al. 2021), DNA methylation analysis has emerged as a method for forensic identification of individuals in a paired MZ twins. In an initial study, Xu et al. reported that DNA methylation at LINE-1 has great potential to discriminate between MZ twins, but its difference was observed in only a small percentage of MZ twins (12.61%) (Xu et al. 2015). Leander Stewart et al. proposed DNA methylation markers at the Alu sequences to identify an MZ twin (Stewart et al. 2015).

DNA microarray analysis is a valuable tool for DNA methylome profiling and discovering new biomarkers. Several researchers have used DNA microarrays to identify MZ twins. Du et al. reported 38 differentially methylated regions in four MZ twins by methylated DNA immunoprecipitation (MeDIP) (Du et al. 2015). Furthermore, Vidaki et al. conducted DNA methylome profiling in 10 pairs of female MZ twins using HumanMethylation 450 K BeadChip and reported twin-differentially methylated sites (Vidaki et al. 2017). Currently, many genome-wide DNA methylome profiling techniques are available, and using more samples for DNA methylome profiling is helpful to identify recurrent DNA methylation markers for discrimination of MZ twins.

To identify potential DNA methylation markers to discriminate MZ twins, we performed genome-wide DNA methylome profiling with 47 paired MZ twin samples using Illumina HumanMethylation EPIC BeadChip, which includes over 850,000 CpG sites. We identified the 26,874–147,713 CpG sites from each pair of MZ twins, and then selected 8,583 recurrent differentially methylated CpG sites (DMCs) between MZ twins. Furthermore, our recurrent DMCs between MZ twins were validated in another cohort from GEO. Our data will be helpful for the development of DNA methylation markers for use in forensic science.

## Materials and methods

### Study subjects and DNA isolation

Blood samples were collected from 94 healthy volunteers in a Korean cohort after obtaining informed consent from the participants. This study was approved by the Institute Review Board of the National Biobank of Korea (https://www.nih.go.kr/biobank/) and Seoul National University Hospital (C-2110-183-1267). Genomic DNA was isolated from blood using DNeasy Blood and Tissue Kit (Qiagen, Carlsbad, CA). The quality and quantity of the extracted genomic DNA were assessed with an ND-1000 spectrophotometer (Nanodrop Technologies, Wilmington, DE).

### DNA methylome profiling and data analysis

HumanMethylation EPIC BeadChip (Illumina, San Diego, CA) was used for DNA methylome screening according to the manufacturer’s instructions. For DNA methylome data analysis, CpG methylation values were calculated as average-β values using the minfi package (version 1.26.2) (Aryee et al. 2014) of R software, and we used the subset-quantile within array normalization (SWAN) to reduce technical variations within and between arrays (Maksimovic et al. 2012). Measurements with detection *P-*values < 0.05 were considered to have a significant signal above the background.

### Public data collection

To validate DMCs selected from our cohort, we obtained a DNA methylation dataset of blood from GEO (GSE154566) generated from 118 paired MZ twin samples using the HumanMethylation EPIC BeadChip platform.

### Motif and gene enrichment analysis

Analysis of sequence motifs was performed using a HOMER package (version 4.10) with the default parameter settings, using recurrent DMCs (Heinz et al. 2010). Regions for motif analyses were defined as 100 bp upstream to 100 bp downstream of DMCs. We used the EnrichR tool (https://maayanlab.cloud/Enrichr/) (Chen et al. 2013) for Kyoto Encyclopedia of Genes and Genomes (KEGG) Gene ontology (GO) enrichment analysis.

### Statistical analysis

R software (version 4.2.2) was used to analyze and plot data. To identify recurrent DMCs between MZ twins, we used standard deviation and methylation differences for each paired MZ twin sample. We employed Student’s *t*-test to evaluate the difference in the number of DMCs between male and female groups. Pearson’s correlation was performed to confirm the association between age and the number of DMCs. The chi-square test was utilized for the DMCs proportion of CpG density or gene category in genomic regions. Results with *P*-values < 0.05 were considered significant.

## Results

### Methylation difference between each paired MZ twin sample

**Fig. 1:**
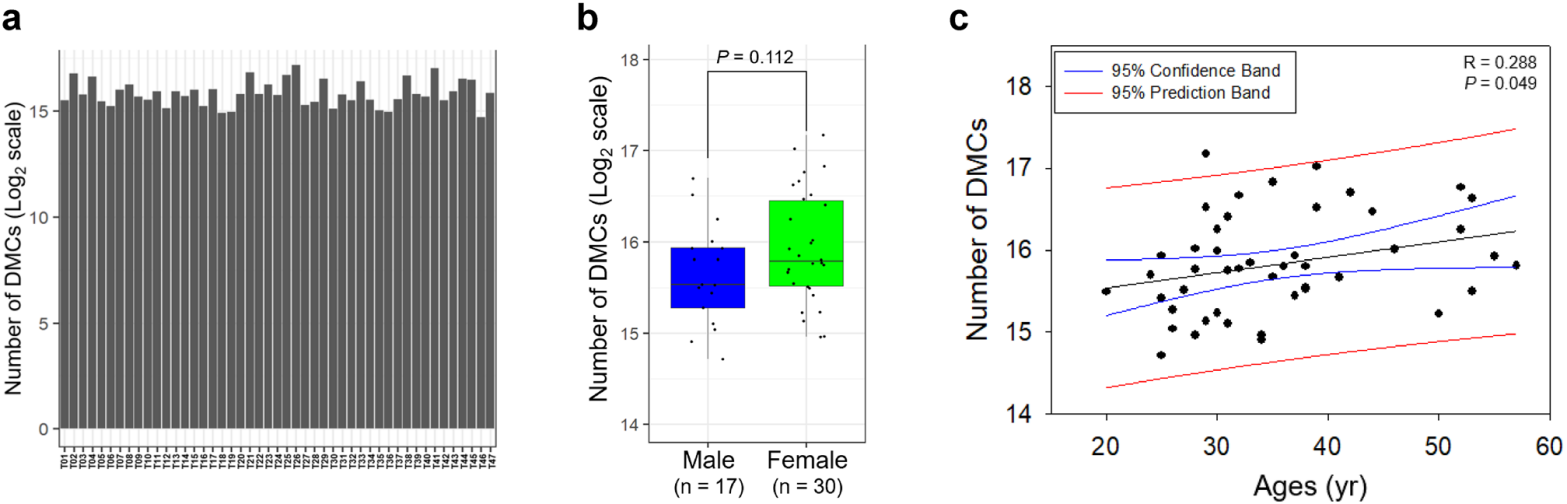
Differentially methylated CpG sites in each paired MZ twin sample. **a** Number of DMCs in each paired MZ twin sample. **b** Difference in the number of DMCs between male and female groups. **c** Correlation between the number of DMCs and age

We carried out DNA methylome profiling using the HumanMethylation EPIC BeadChip platform of blood collected from 47 paired MZ twin samples. For identification of DMCs between paired MZ twin samples, we calculated the methylation difference for each paired MZ twin sample. DMCs with methylation difference values higher than 0.05 (average-β values) were considered. As a result, we identified 26,874–147,713 CpG sites from each paired MZ twin sample (Fig. 1a). The number of DMCs was not significantly different between male and female groups (Fig. 1b), but it did increase with age (Fig. 1c).

### Identification of recurrent DMCs and their characteristics

**Fig. 2:**
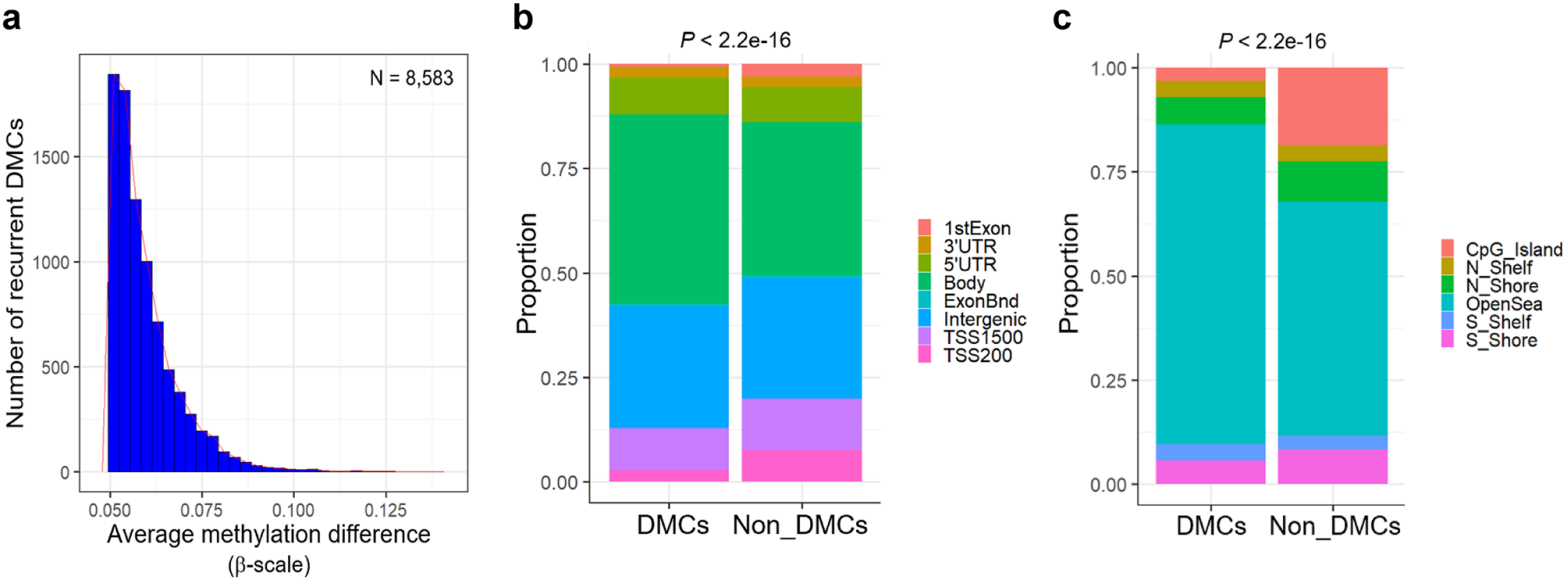
Distribution of recurrent DMCs between MZ twins and their proportion of CpG density of gene category in genomic regions. **a** Distribution of recurrent DMCs with average methylation differences. **b** genomic distribution, **c** CpG relationship. ExonBnd, within 20 bases of an exon boundary; Shore, ~ 0–2 kb from the CGI; shelf, ~ 2–4 kb from the CGI; open sea, > 4 kb from the CGI. CGI, CpG island

For identification of recurrent DMCs between paired MZ twin samples, we applied the following two criteria: (1) DNA methylation standard deviation > 0.05 (average-β values) and (2) average methylation difference > 0.05 (average-β values) between MZ twins. As a result, we identified 8583 recurrent DMCs (Fig. 2a). We analyzed the distribution feature of recurrent DMCs based on the gene structure and CpG island relationship. Gene structures were classified into eight categories: TSS1500, TSS200, 1stExon, body, intergenic, ExonBnd, 3’UTR, and 5’UTR. Our results showed that recurrent DMCs were significantly enriched in the gene body regions compared to the non-DMCs (Fig. 2b). CpG island relationships were classified into four categories: island, shore (up to 2 kb from CpG island), shelf (2–4 kb from CpG island), and open sea (> 4 kb from CpG island). We observed that recurrent DMCs were significantly enriched in open seas compared to non-DMCs (Fig. 2c).

### Recurrent DMCs associated with the immune system

**Fig. 3:**
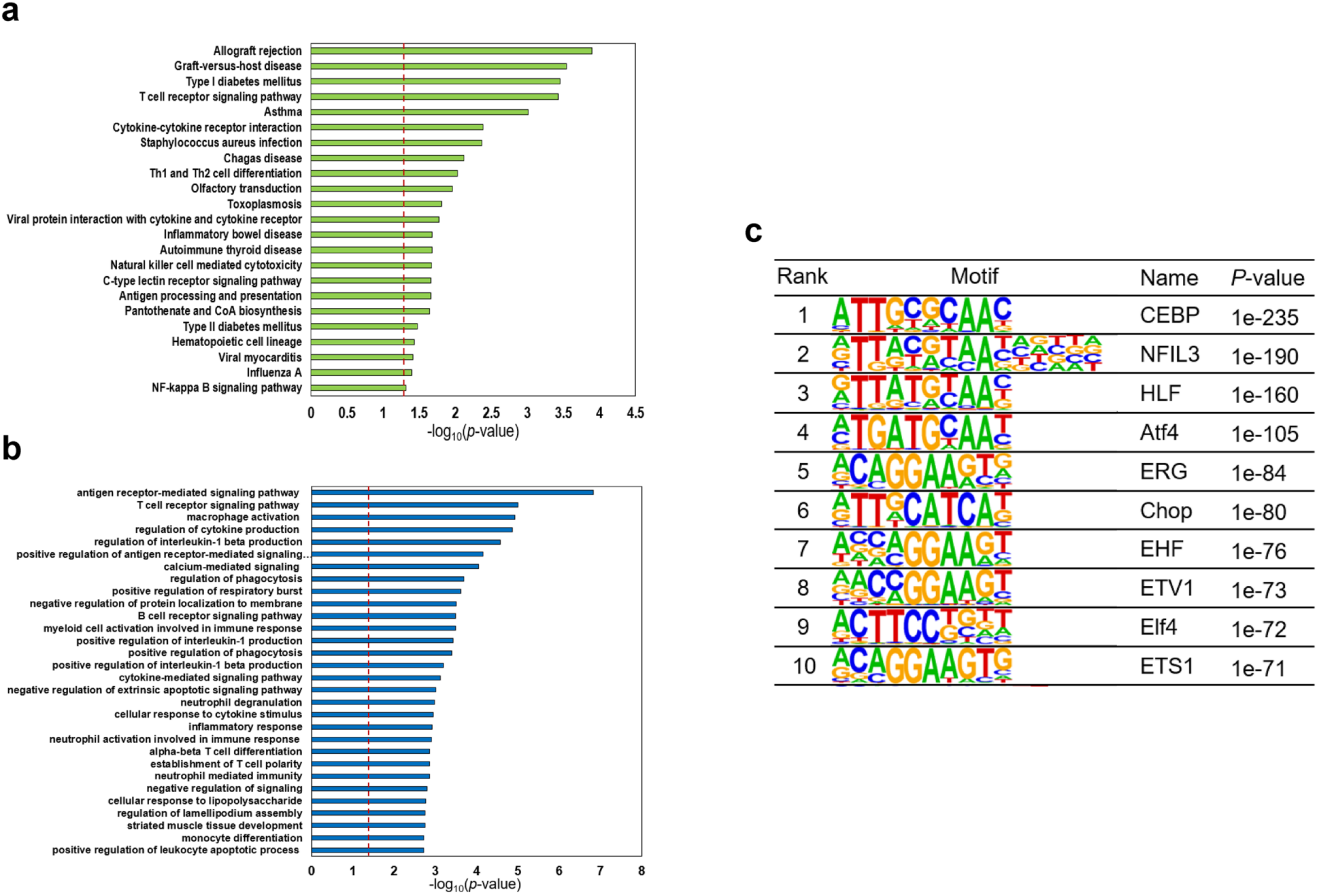
Enrichment analysis using recurrent DMCs between MZ twins. **a** GO and **b** KEGG pathway analyses using the EnrichR tool. For GO and KEGG enrichment analyses, 1210 recurrent DMCs located at TSS were used. **c** Recurrent DMCs of known transcription factor binding sites based on HOMER packages with default parameter settings. Sequences within + 100 bp or − 100 bp flanking each of the recurrent DMCs were used for known motif analyses

We performed KEGG pathway, GO, and motif analyses to reveal the biological function of recurrent DMCs in transcription start site (TSS) regions. KEGG pathway analysis revealed allograft rejection, T-cell receptor signaling pathway, asthma, and Th1 and Th2 cell differentiation (Fig. 3a). In GO analysis, the antigen receptor-mediated signaling pathway, T-cell receptor signaling pathway, macrophage activation, and regulation of cytokine production were highlighted (Fig. 3b). Transcription factors are proteins with DNA binding activity that are involved in regulation of transcription. In generally, TFs modulate gene expression by binding to gene promoter regions of distal regions called enhancers. The distance between a TFBS and a TSS of a gene regulated by a TF can be up to several megabases, and depends on the chromatin structures of the regions (Dekker and Heard 2015). To examine whether the recurrent DMCs identified are associated with TFBSs, we performed TF motif analysis using the 8,583 recurrent DMCs. Our results revealed the recurrent DMCs to be enriched in CEBP, NFIL3, HLF, Atf4, and ERG and involved in regulation of the immune system (Kim et al. 2019; Ng et al. 2020; Poli 1998; Yang et al. 2018). The top 10 binding motifs for the recurrent DMCs are shown in Fig. 3c. These results indicated that the recurrent DMCs are related to the immune system.

### Validation of recurrent DMC in the public dataset

**Fig. 4:**
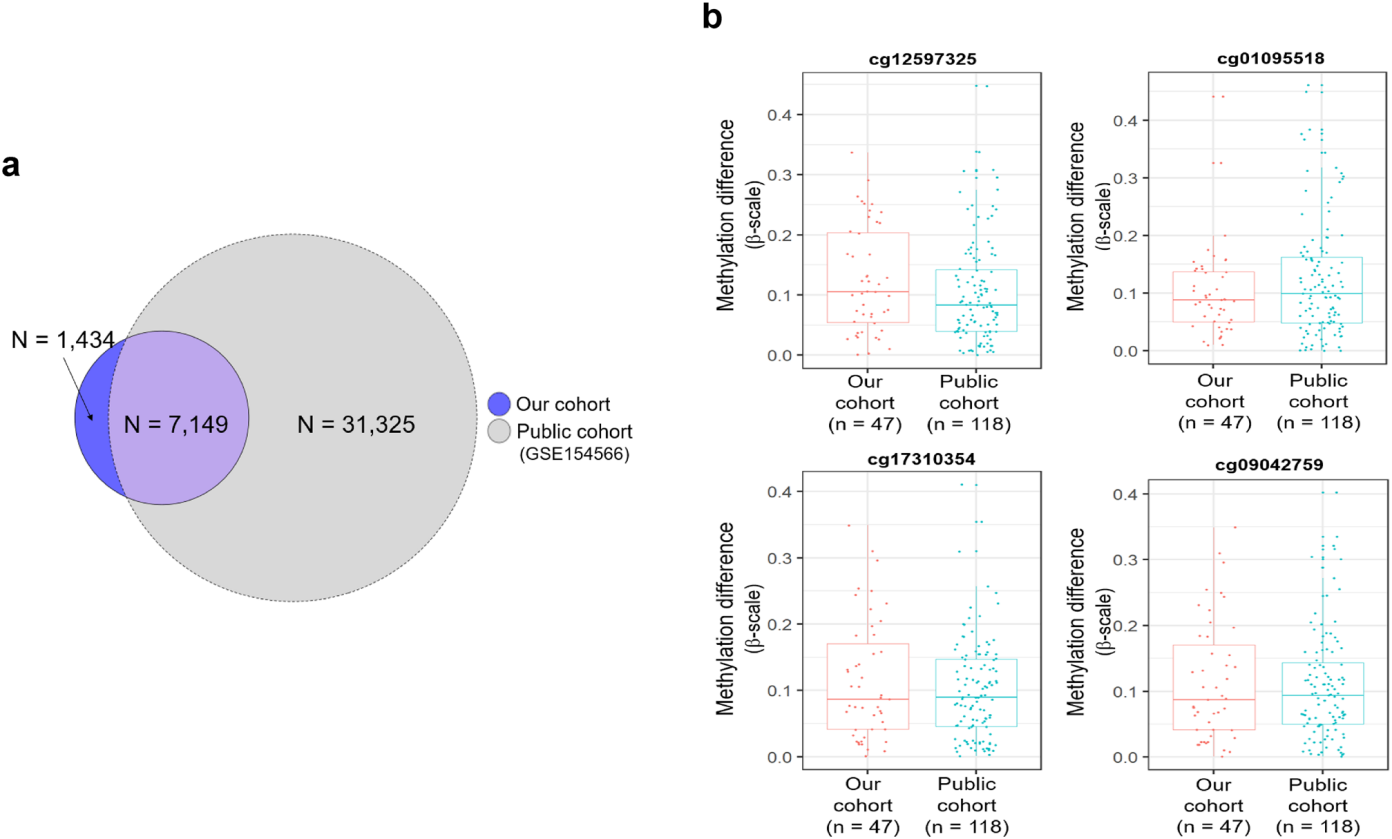
Validation of our recurrent DMCs in a public dataset. **a** Venn diagram of recurrent DMCs identified from our cohort and public cohort (GSE154566). **b** Methylation difference levels in each MZ paired twin sample of the top 4 candidate DNA methylation markers in our and public cohorts

To validate our recurrent DMCs, we collected DNA methylome data from the GEO public database and then analyzed the public methylome data in the same way. Of our 8583 recurrent DMCs, 7149 were validated in the public dataset (Fig. 4a). The methylation difference in each paired MZ twin samples of the top 4 recurrent DMCs (cg12597325, cg01095518, cg17310354, and cg09042759) is shown in Fig. 4b. The top 20 candidate recurrent DNA methylation markers to discriminate between MZ twins are summarized in Table 1. The results suggest that DNA methylation has a great potential application for forensic identification of individuals in an MZ twin pair.

**Table 1:** Top 20 candidate DNA methylation markers to discriminate between MZ twins

Illumina ID^a^	Average delta-β		Chromosome	MapInfo^b^	Symbol	Gene location	CpG island^c^
	Our dataset	Public dataset					
cg12597325	0.125	0.104	chr12	131281504	STX2	Body	OpenSea
cg01095518	0.101	0.123	chr6	655849	EXOC2	5'UTR	N_Shore
cg17310354	0.122	0.101	chr11	18260736	SAA2	3'UTR	OpenSea
cg09042759	0.113	0.109	chr6	32134176	EGFL8	Body	N_Shore
cg12273505	0.103	0.118	chr10	35534954	CCNY	TSS1500	OpenSea
cg06354455	0.117	0.091	chr13	114054873			N_Shore
cg04804472	0.104	0.101	chr3	21267468			OpenSea
cg04373893	0.097	0.103	chr15	42785639			S_Shelf
cg12482564	0.099	0.098	chr4	170703951			OpenSea
cg05060085	0.097	0.097	chr2	672793	TMEM18	Body	N_Shelf
cg15884546	0.091	0.103	chr12	9822213	CLEC2D	TSS200	OpenSea
cg02160684	0.095	0.096	chr8	126448033	TRIB1	Body	S_Shelf
cg09126794	0.097	0.094	chr8	1879551	ARHGEF10	Body	OpenSea
cg08810073	0.091	0.099	chr20	33214972	PIGU	Body	OpenSea
cg13650965	0.097	0.092	chr6	41011991	TSPO2	3′UTR	OpenSea
cg13826058	0.095	0.094	chr6	125626391			S_Shelf
cg19005485	0.092	0.095	chr3	197272328	BDH1	Body	OpenSea
cg23042540	0.094	0.091	chr8	1653566	DLGAP2	3′UTR	S_Shelf
cg22683377	0.092	0.092	chr8	28371364	FZD3	Body	OpenSea
cg13027965	0.091	0.093	chr6	144056673	PHACTR2	Body	OpenSea

^a^Illumina ID is the unique identification number in HumanEPIC BeadChip

^b^Mapinfo indicates the genomic location in human reference genome 37 (GRCh37/hg19), as released by the Genome Reference Consortium on March 3, 2009 (www.ncbi.nlm.nih.gov/projects/genome/assembly/grc/human/)

^c^Shore and shelf are adjacent to the CpG island (2- and 4-kb regions flanking the CpG island, respectively). N and S indicate upstream and downstream of the CpG island, respectively

## Discussion

Tissue-specific patterns of DNA methylation have been demonstrated (Kitamura et al. 2007; Song et al. 2009). In this study, we chose to base our analysis on blood samples because blood is commonly found at crime scenes. To identify potential DNA methylation markers for identifying an individual in a pair of MZ twins, we conducted genome-wide DNA methylation profiling with 47 paired MZ twin samples using HumanMethylaton EPIC BeadChip. Then, we observed tens to hundreds of thousands of DMCs for each individual paired MZ twin samples. The change in DNA methylation between MZ twins was not significantly different between male and female groups, but it correlated with age. Our result is similar to previous results (Kim et al. 2018; Martin 2005).

The immune system specializes in responding to environmental exposures (MacGillivray and Kollmann 2014), and DNA methylation plays an important role in the immune system by controlling gene expression through changes in chromatin structure(Morales-Nebreda et al. 2019). KEGG and GO analyses showed recurrent DMCs between MZ twins to be enriched in the immune system. Enhancers or superenhancers (Sur and Taipale 2016) are also associated with DNA methylation (Heyn et al. 2016), and our motif analysis showed the recurrent DMCs to be enriched at immune system-associated transcription factor-binding sites such as CEBP (Larabee et al. 2018), NFIL3 (Schlenner et al. 2019), and ATF (Jadhav and Zhang 2017).

Several techniques are available for measuring DNA methylation levels (Laird 2010). In this study, we identified recurrent DMCs, but the difference in methylation level of most recurrent DMCs was less than 0.1 (βvalues). Therefore, it is essential to choose a validation method for DNA methylation with few technical errors. Further study will be needed to validate our recurrent DMCs by validation techniques such as pyrosequencing (Ronaghi 2001) and amplicon bisulfite sequencing (Masser et al. 2013), as these techniques provide high-accuracy methylation measurements and require small amounts of DNA.

In conclusion, we expect that our DNA methylation study and novel DNA methylation markers will help to improve the use of DNA to discriminate individuals in a pair of monozygotic twins.

## Data Availability

All primary methylation array data were deposited in the GEO (https://www.ncbi.nlm.nih.gov/geo/) public database under Accession Number GSE225544.
